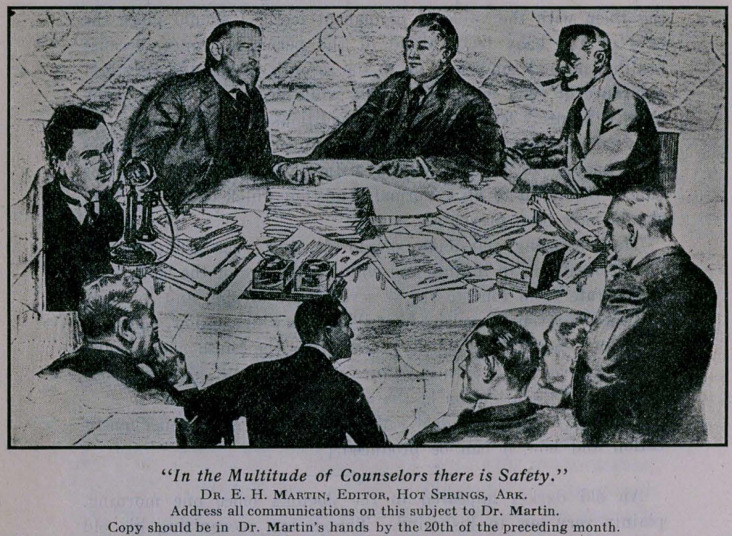# Pellagra Forum

**Published:** 1916-10

**Authors:** 


Our only paper on pellagra this month is by Dr. Duane Meredith, the new Professor of Embryology, Histology and Research Medicine in the Medical Department of the T. C. U. University of Fort Worth.

Dr. Meredith's paper is especially valuable in calling attention to early symptoms. Some of these have not been mentioned before, especially pustular eruptions in the hair of early cases. We have not observed this, but probably have not seen the cases early enough. In some older cases furunculosis has been a very prominent symptom.

One early symptom which Dr. Meredith does not mention is the increased patellar reflexes, in fact inclination toward exaggeration of all reflexes. Sometimes this is one of the very earliest symptoms. Another is a tendency for the gums to bleed when the patient uses a toothbrush. This is a very common symptom even when not accompanied by a red tongue and stomatitis.

The description given of the condition of the bowels is very accurate. A good many patients having pellagra will give a history of having had an operation for hemorrhoids the summer before the diagnosis was made. This in most of those cases was evidently the first symptom of pellagra, probably not a true hemorrhoidal condition but more of a proctitis.

It is to be hoped that Dr. Meredith will solve the problem of the cause of pellagra, but many a germ has been watched so far without results.—E. H. M.

## Figures and Tables

**Figure f1:**